# S141. TRANSGENIC OVEREXPRESSION OF THE TYPE III ISOFORM OF NEUREGULIN 1 IN MICE INDUCES ABNORMALITIES ON AUDITORY EVENT RELATED EEG BIOMARKERS RELATED TO SCHIZOPHRENIA

**DOI:** 10.1093/schbul/sby018.928

**Published:** 2018-04-01

**Authors:** Holger Rosenbrock, Wiebke Nissen, Roberto Arban, Rossner Moritz, Schwab Markus, Cornelia Dorner-Ciossek, Niklas Schülert

**Affiliations:** 1 Boehringer Ingelheim Pharma GmbH & Co KG; 2 CNS Diseases Research, Boehringer Ingelheim Pharma GmbH & Co KG; 3 Ludwig-Maximilians-Universität München; 4 Hannover Medical School

## Abstract

**Background:**

Genetic, post-mortem and preclinical studies in transgenic mice repeatedly implicate neuregulin 1 (NRG1) as a critical component in the pathophysiology of schizophrenia. Its predominant neuronal receptor, ErbB4, is primarily expressed in fast-spiking interneurons enabling the maintenance of normal excitatory/inhibitory balance (E/I balance) of neuronal networks. Changes in E/I balance can be assessed in-vivo via special electroencephalography (EEG) techniques and have become an important preclinical and clinical readout to investigate the underlying mechanisms of psychiatric disorders. In fact, patients with schizophrenia show aberrant processing of sensory information leading to deficits in auditory event-related potentials (AERP), the detection of deviant auditory stimuli (mismatch negativity, MMN) and the 40Hz auditory steady-state response (ASSR) as well as to increased basal gamma oscillation.

Patients with Schizophrenia carrying NRG1 HapICE risk alleles appear to overproduce the NRG1 type III isoform in their brain. In the transgenic mouse, NRG1 type III overexpression (HANI mice; Velanac et al., 2012) results in altered synaptic activity and in behavioural changes like reduced prepulse inhibition and impaired cognition compatible with a schizophrenia-related phenotype (Agarwal et al, 2014). In the present study, the potential disruption of the E/I balance in HANI mice has been investigate via EEG recording.

**Methods:**

Superficial electrodes were implanted above the auditory cortex and the frontal cortex. We used a novel wireless neurologger system for the recording of EEG data in awake freely moving mice. Data analysis was performed with commercially available software which is also used in clinical setting.

**Results:**

Overexpression of NRG1 abolished MMN, significantly increased the P1 and N1 amplitude of AERP, increased basal gamma oscillation and reduced phase-lock coherence in the 40 Hz ASSR compared to the wildtype littermates.

**Discussion:**

In this study we showed for the first time that overexpression of NRG1 leads to deficits in event-related EEG biomarkers supporting the notion that the NRG1-ErbB4 pathway is involved in maintaining the E/I balance, sensory stimulus processing and ultimately cognitive function. Our results indicate that the NRG1 III tg mouse model represents a tool with high translational potential to investigate pathological mechanisms related to schizophrenia.

